# Surfactin from *Bacillus subtilis* induces apoptosis in human oral squamous cell carcinoma through ROS-regulated mitochondrial pathway

**DOI:** 10.7150/jca.50835

**Published:** 2020-10-21

**Authors:** Thi Thuy Tien Vo, Ju-Fang Liu, Ching-Zong Wu, Wei-Ning Lin, Yuh-Lien Chen, I-Ta Lee

**Affiliations:** 1 School of Dentistry, College of Oral Medicine, Taipei Medical University, Taipei, Taiwan.; 2 School of Oral Hygiene, College of Oral Medicine, Taipei Medical University, Taipei, Taiwan.; 3 Graduate Institute of Biomedical and Pharmaceutical Science, Fu Jen Catholic University, New Taipei City, Taiwan.; 4 Department of Anatomy and Cell Biology, College of Medicine, National Taiwan University, Taipei, Taiwan.

**Keywords:** apoptosis, surfactin, particulate matter, oral squamous cell carcinoma, reactive oxidative species

## Abstract

Recently, ambient air particulate matter (PM) has been shown to increase the risk of oral cancer. The most common malignant tumor in the oral cavity is oral squamous cell carcinoma (OSCC). Recent studies have revealed that surfactin, a cyclic lipopeptide generated by *Bacillus subtilis*, has anti-inflammatory and anti-cancer properties. However, the exact anti-cancer effects of surfactin on human OSCC and underlying molecular mechanisms remain largely unknown. In the present study, we found that treatment of SCC4 and SCC25 cells (human OSCC cell lines) with surfactin reduced the viability of SCC4 and SCC25 cells by induction of apoptosis. Surfactin-induced apoptosis was associated with caspase activation and poly(ADP-ribose) polymerase (PARP) cleavage and was regulated by the mitochondrial pathway, exemplified by mitochondrial depolarization, mitochondrial-derived reactive oxidative species (ROS) production, cytochrome c release, up-regulation of Bad and Bax, and down-regulation of Bcl-2. Surfactin induced NADPH oxidase-dependent ROS generation, which appeared essential for the activation of the mitochondrial pathway. Surfactin-induced mitochondrial-derived ROS generation was associated with JNK1/2 activation. After treatment with surfactin, ROS caused JNK1/2-dependent cell death of SCC4 and SCC25 cells. Taken together, our findings suggest that surfactin induces mitochondria associated apoptosis of human OSCC cell lines, and surfactin may be a potential chemotherapeutic agent for future OSCC treatment.

## Introduction

In many parts of the world, new cases of oral cancer and deaths are increasing. Known risk factors include smoking, drinking, human papillomavirus (HPV) and betel quid chewing 1. It is additionally believed that exposure to heavy metals, as well as emissions from petroleum and chemical plants, is also related to the development of oral cancer. To date, it is well known that air pollution, especially ambient air particulate matter (PM), is harmful to the respiratory and cardiovascular system 2. The combined effects of household and ambient air pollution cause approximately 7 million premature deaths every year, mainly due to heart disease, stroke, lung cancer, chronic obstructive pulmonary disease, and acute respiratory infections, leading to increased mortality 3,4. The composition of PM is highly complicated, including nitrate, sulfate, ammonia, and so on. Compared to PM10, PM2.5 can cause greater damage to human health. PM2.5 generally penetrates the lung barrier and enters the blood system. Most studies in the past had explored the relationship between betel nuts, cigarettes, or alcohol consumption and oral cancer, but few investigations have examined the relationship between air pollution and oral cancer. The most common malignant tumor in the oral cavity is oral squamous cell carcinoma (OSCC). Despite considerable advances in cancer treatment, surgical resection followed by adjuvant radiation therapy and chemotherapy remains the conventional regimen that is widely accepted in this field. Besides, a variety of new treatment strategies, including immunotherapy, nanotechnology, and molecular-targeted therapy, has emerged as potential options, but still display limited success without substantial clinical effect 5-7.

Natural products have the characteristics of safety and uncommon side effects. Therefore, they are increasingly used as a positive source for development of novel cancer preventive and therapeutic drugs 8,9. For instances, curcumin, resveratrol, apigenin, eugenol, and genistein are well-known cancer preventive agents, and some of their analogues have synergic effects targeting multiple pathways, including cyclooxygenase-2 (COX-2), signal transducer and activator of transcription-3 (STAT-3) signaling, and reactive oxidative species (ROS) 10-14. Among them, surfactin is a bacterial cyclic lipopeptide generated by *Bacillus subtilis* 15. Recently, surfactin has been shown to possess some properties including anti-cancer, anti-bacterial, and anti-viral activities 16. Park et al. indicated that surfactin could reduce 12-O-tetradecanoylphorbol-13-acetate (TPA)-mediated breast cancer cell migration/invasion via the inhibition of matrix metallopeptidase-9 (MMP-9) levels 15. In addition, Wang et al. also proved that surfactin could promote apoptosis of HepG2 cells via the ROS signaling 17. Despite its various activities, the molecular basis behind the pharmacological activities of surfactin and its therapeutic effects on OSCC remain largely unknown. Here, we studied the anti-cancer effects of surfactin on PM-treated human OSCC and the novel mechanisms underlying these processes. In the present study, we report that the pro-apoptotic effect of surfactin in SCC4 and SCC25 human OSCC cell lines is regulated by increasing ROS production, activation of the JNK1/2 phosphorylation cascade, and the mitochondrial pathways. This study therefore provides evidence, for the first time, that surfactin can be considered as a potential chemotherapeutic agent for the treatment of human OSCC.

## Methods

### Materials

Anti-GAPDH, anti-phospho-JNK1/2, anti-β-actin, anti-caspase-3, anti-caspase-7, anti-caspase-9, anti-poly(ADP-ribose) polymerase (PARP), anti-Bax, anti-Bad, anti-Bcl-2, anti-cytochrome c, anti-Akt, and anti-COX IV antibodies were obtained from Santa Cruz Biotechnology, Inc. (Santa Cruz, CA). SP600125, Z-VAD-FMK, MitoTEMPO, surfactin, and urban PM (SRM 1648a) were purchased from Sigma (St. Louis, MO, USA). N-acetyl-L-cysteine (NAC) and diphenyleneiodonium chloride (DPI) were taken from Biomol (Plymouth Meeting, PA).

### Cell culture

SCC4 and SCC25 human OSCC cell lines were kindly provided by Dr. J. F. Liu (School of Oral Hygiene, College of Oral Medicine, Taipei Medical University, Taipei, Taiwan). SCC4 and SCC25 cells were grown in DMEM/F12 supplemented with 10% fetal bovine serum (FBS), 2 mM glutamine and 0.4 μg/ml hydrocortisone. Cells were maintained as monolayer cultures in a humidified atmosphere of 5% CO_2_ at 37°C. In addition, human gingival fibroblasts (HGFs) and human oral keratinocytes (HOKs) were purchased from ScienCell Research Laboratories (San Diego, CA). They were cultured in fibroblast medium (ScienCell Research Laboratories, San Diego, CA) and oral keratinocyte medium (ScienCell Research Laboratories, San Diego, CA), respectively, supplemented with 10% FBS, 100 μg/ml streptomycin, and 100 U/ml penicillin at 37°C in 5% CO_2_. Experiments were performed with cells from passages 3 to 8. Prior to treatment or stimulation with reagents, cells were serum starved for 24 h.

### Cell viability

The cell viability of SCC4 and SCC25 cells in response to surfactin was assessed using PrestoBlue Cell Viability Reagent (Invitrogen, CA, USA) according to the manufacturer's protocol.

### Western blot

SCC4 and SCC25 cells were grown to confluence in 6-well plates and then treated with surfactin for the indicated time intervals. The cells were washed, scraped, collected, and centrifuged at 45000 × *g* at 4°C for 1 h to yield the whole cell extract, as previously described 18. Samples were denatured, subjected to SDS-PAGE using a 12% running gel, and transferred to nitrocellulose membrane. Membranes were incubated with anti-caspase-3, anti-caspase-7, anti-caspase-9, anti-PARP, anti-Bcl-2, anti-Bax, anti-Bad, or anti-phospho-JNK1/2 antibody for 24 h, followed by incubation with anti-mouse or anti-rabbit horseradish peroxidase antibody for 1 h. The immunoreactive bands were detected by ECL reagents developed by Hyperfilm-ECL.

### Caspase activity determinations

Caspase activity in cell lysates was measured using the manufacturer's protocols (caspase-3, -7, and -9 colorimetric assay kits; R&D Systems Inc., Minneapolis, MN, USA). Cells were treated with surfactin for 48 h and then lysed in lysis buffer [50 mM Tris-HCl (pH 7.4), 1 mM EDTA, 10 mM EGTA, 10 mM digitonin, and 2 mM DTT]. The cell lysates (50 μg proteins) were incubated with caspase-3, -7, and -9 specific substrates (Ac-DEVD-pNA and Ac-LEHD-pNA) at 37°C for 1 h. Caspase activity and absorbance were measured with an enzyme-linked immunosorbent assay reader at OD_405_. All results were obtained from three independent experiments.

### Cytosolic and mitochondrial protein extraction

To obtain cytosolic and mitochondrial fractions, cells were treated with a digitonin buffer (20 mM Hepes-KOH, pH 7.3, 110 mM KAc, 5 mM NaAc, 2 mM MgAc2, 1 mM EGTA, and 200 μg/ml digitonin) on ice for 10 min to permeabilize the cell membrane. The cell lysate was then centrifuged at 10000 × *g* at 4°C for 15 min. The supernatant was collected as a cytosolic fraction, and the pellet (mitochondria-containing fraction) was resuspended in 1X-SDS-loading buffer. Protein content was estimated according to a commercial protein assay (Bio Rad, Milan, Italy), and the samples were either analyzed immediately or stored at -80°C. Total, cytosolic, and mitochondrial extracts were then analyzed by Western blot.

### Mitochondrial membrane potential detection

Mitochondrial membrane potential (ΔΨm) was detected by a fluorescent dye JC-1 (Sigma, St. Louis, MO). The change from red fluorescence to green fluorescence in the JC-1 assay can be used to detect the decline in mitochondrial membrane potential. Furthermore, this transition can also be used as an early detection indicator of apoptosis. After being treated with various concentrations of surfactin for 48 h, the SCC4 cells in 6-well plate were washed with PBS twice, and then 1 ml of serum-free DMEM/F-12 medium was added followed by 1 ml of JC-1 staining working solution in each well. The plate was incubated for 20 min in the incubator at 37°C with 5% CO_2_. The plate was observed and photographed under a fluorescence microscope (Carl Zeiss, Gottingen, Germany). The wavelengths of excitation and emission were 514 nm and 529 nm for detection of JC-1 monomers, respectively. The values of 585 nm and 590 nm were used to detect JC-1 aggregates. The relative ratio of red and green fluorescence represented the change of mitochondrial membrane potential (ΔΨm). Five groups of data of each well were recorded.

### Determination of NADPH oxidase activity by chemiluminescence assay

After incubation, cells were gently scraped and centrifuged at 400 × *g* for 10 min at 4°C. The cell pellet was resuspended with 35 μl of ice-cold RPMI-1640 medium per well, and the cell suspension was kept on ice. To a final 200 μl volume of pre-warmed (37°C) RPMI-1640 medium containing either NADPH (1 μM) or lucigenin (20 μM), 5 μl of cell suspension (0.2 × 10^5^ cells) were added to initiate the reaction followed by immediate measurement of chemiluminescence in an Appliskan luminometer (Thermo^®^) in out-of-coincidence mode. Appropriate blanks and controls were established, and chemiluminescence was recorded. Neither NADPH nor NADH enhanced the background chemiluminescence of lucigenin alone (30-40 counts per min). Chemiluminescence was continuously measured for 12 min, and the activity of NADPH oxidase was expressed as counts per million cells.

### Measurement of intracellular ROS and mitochondrial ROS generation

CellROX Green Reagent and MitoSOX Red mitochondrial superoxide indicator (Molecular Probes, Eugene, OR) were used in these experiments. For the purpose of these experiments, SCC4 cells were washed with warm Hank's Balanced Salt Solution (HBSS) and incubated in HBSS or cell medium containing 5 μM CellROX Green Reagent or MitoSOX Red mitochondrial superoxide indicator at 37°C for 30 min. Subsequently, HBSS or medium containing CellROX Green Reagent or MitoSOX Red mitochondrial superoxide indicator was removed and replaced with fresh medium. SCC4 cells were then incubated with surfactin for the indicated times. Cells were washed twice with PBS and detached with trypsin/EDTA, and the fluorescence intensity of the cells was analyzed using a FACScan flow cytometer (BD Biosciences, San Jose, CA) at 485 nm excitation and 520 nm emission (CellROX Green Reagent) and 510 nm excitation and 580 nm emission (MitoSOX Red mitochondrial superoxide indicator), respectively.

### Transient transfection with human siRNAs

Human scrambled, JNK1, and JNK2 siRNAs were taken from Santa Cruz Biotechnology Inc (Santa Cruz, CA, USA). Transient transfection of siRNAs was performed using a Lipofectamine 2000 Transfection Reagent (Invitrogen, CA, USA) according to the manufacturer's instructions.

### DAPI staining

SCC4 and SCC25 cells (5000 cells/ml) in 24-well plates were incubated with surfactin (15 or 30 μM) for the indicated times. Cells in each treatment were individually fixed with 3.7% (vol/vol) formaldehyde for 15 min and then stained by DAPI (0.5 μg/ml) for determining cell chromatin condensation. All samples were examined and photographed using Nikon Eclipse TE300 inverted fluorescence microscope (Nikon Corp. Tokyo, Japan).

### Statistical analysis

We analyzed the data with the GraphPad Prism program (GraphPad, San Diego, CA, USA). Quantitative data were expressed as the mean±S.E.M. and analyzed with one-way ANOVA followed with Tukey's post-hoc test. We considered *P*<0.05 as a significant difference.

## Results

### Surfactin decreases cell viability and induces apoptosis in SCC4 and SCC25 cells

**Figure 1:**
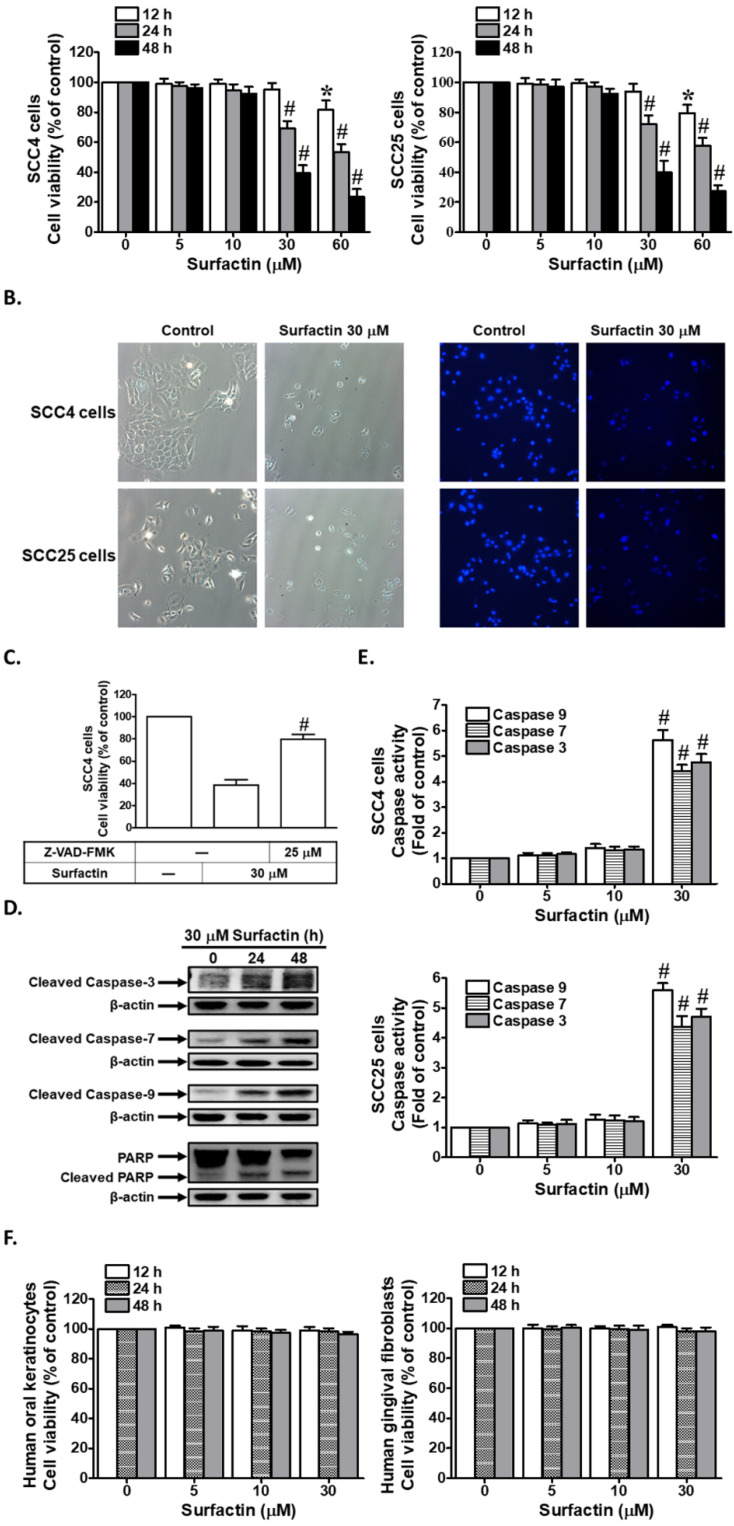
** Surfactin reduces cell viability and induces apoptosis in SCC4 and SCC25 cells. (A)** Cells were treated with various concentrations of surfactin for the indicated times and then the cell viability was measured. **(B)** Cells were treated with 30 μM surfactin for 48 h and then the occurrence of apoptosis was evaluated by DAPI staining. **(C)** SCC4 cells were pretreated with Z-VAD-FMK for 1 h and then incubated with surfactin for 48 h. Cell viability was assayed. **(D)** SCC4 cells were treated with surfactin for 24 or 48 h and then the cleaved caspase-3, -7, -9 and PARP protein levels were determined.** (E)** Cells were treated with various concentrations of surfactin for 48 h and then the caspase activity was analyzed. **(F)** Human oral keratinocytes and human gingival fibroblasts were treated with various concentrations of surfactin for the indicated times and then the cell viability was assayed. Data are expressed as mean±S.E.M. of four independent experiments. *P < 0.05; #*P* < 0.01, as compared with control (A, E). #*P* < 0.01, as compared with the cells exposed to surfactin alone (C).

To examine the anti-cancer potential of surfactin on human OSCC, its effect was investigated on cell viability using the SCC4 and SCC25 human OSCC cell lines. Cells were treated with different concentrations of surfactin for the indicated times and then cell viability was assessed. As shown in Fig. 1A, surfactin markedly reduced cell viability in SCC4 and SCC25 cells in a concentration- and time-dependent manner. In addition, we also observed that surfactin could decrease cell viability in human osteosarcoma cell lines (HOS and U2OS) (Supplementary Fig. 1). These data suggest that surfactin may cause apoptosis in many cancer cells. On the other hand, the occurrence of apoptosis was evaluated by DAPI staining (Fig. 1B). Caspases play a central role in mediating most apoptotic responses 14. To analyze the apoptotic pathway and the role of caspases involved in apoptosis of SCC4 cells regulated by surfactin, cell viability in SCC4 cells was examined in the presence of the pan-caspase inhibitor Z-VAD-FMK. As shown in Fig. 1C, 30 μM surfactin significantly reduced cell viability in SCC4 cells. Moreover, this observed effect by surfactin was abrogated in the presence of Z-VAD-FMK. This result demonstrated that apoptosis is associated with caspase activation. Caspase-9 is a highly specific protease that only cleaves a few proteins, whereas caspase-3 and caspase-7 contribute to the majority of cleavage that takes place during apoptosis 19. Western blot results of the expression levels of caspase-3, -7, and -9 after exposure to surfactin for different times (Fig. 1D) showed the involvement of these effector caspases. Expression of PARP, a protein targeted and cleaved by caspases during apoptosis, was also examined. As shown in Fig. 1D, surfactin induced PARP cleavage in SCC4 cells. Moreover, in this study, we investigated the effects of surfactin on caspase-3, -7, and -9 activity in SCC4 and SCC25 cells. As shown in Fig. 1E, 30 μM surfactin markedly increased caspase-3, -7, and -9 activity. Finally, we investigated the effects of surfactin on cell viability of normal HOKs and HGFs. Interestingly, as shown in Fig. 1F, surfactin did not affect cell viability of these cells. Taken together, these data suggest that surfactin can decrease cell viability and induce apoptosis in SCC4 and SCC25 cells, which is regulated via caspase activation and PARP cleavage.

### Surfactin induces ROS-dependent apoptosis

**Figure 2:**
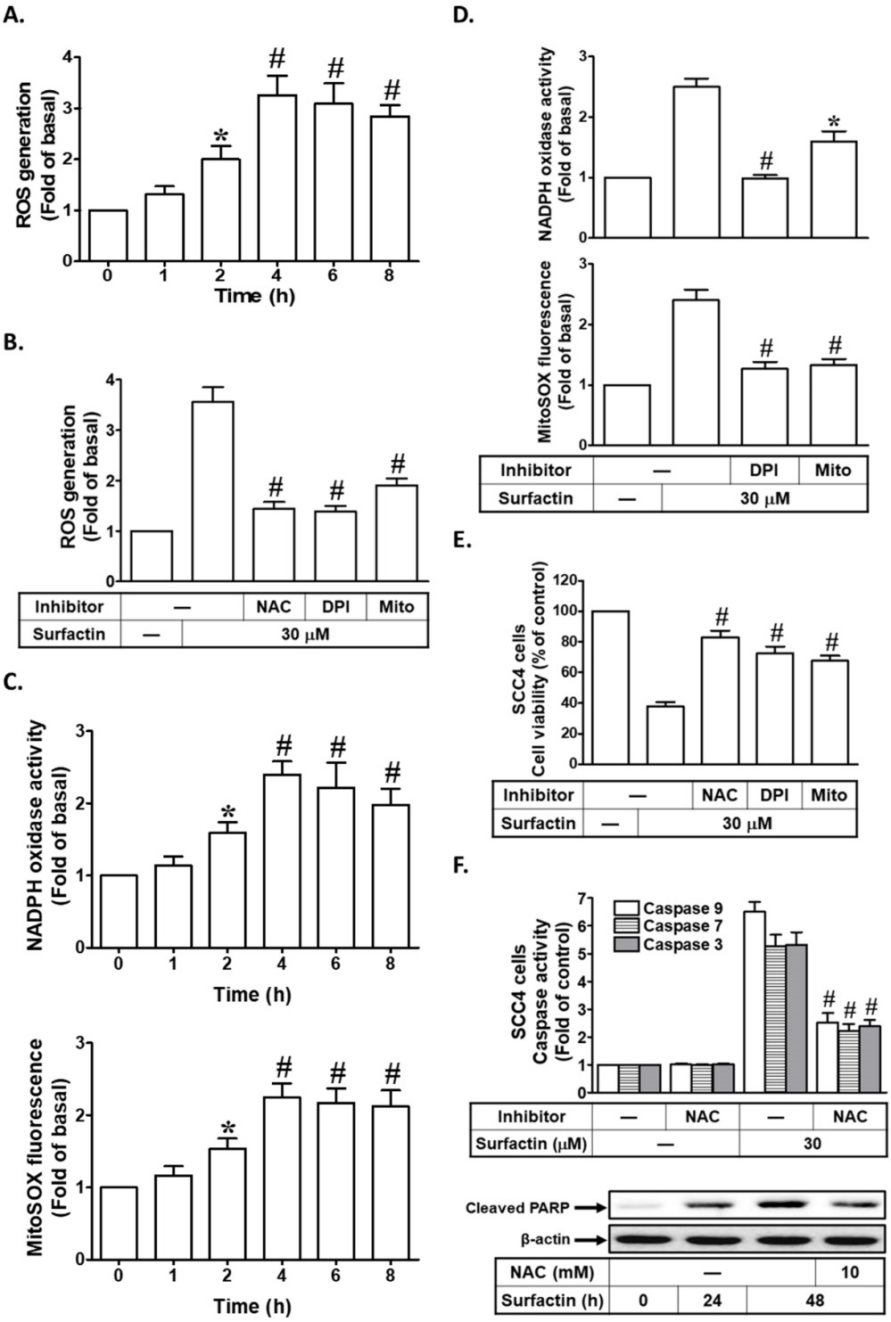
** Surfactin induces ROS-dependent apoptosis. (A)** SCC4 cells were treated with surfactin (30 μM) for the indicated times and then the ROS generation was measured. **(B)** SCC4 cells were pretreated with NAC (10 mM), DPI (1 μM), or MitoTEMPO (10 μM) and then incubated with surfactin for 4 h. The ROS generation was measured. **(C)** SCC4 cells were treated with 30 μM surfactin for the indicated times and then the NADPH oxidase activity and MitoSOX fluorescence were measured. **(D)** SCC4 cells were pretreated with DPI (1 μM) or MitoTEMPO (10 μM) for 1 h and then incubated with surfactin for 4 h. The NADPH oxidase activity and MitoSOX fluorescence were measured. **(E)** Cells were pretreated with NAC (10 mM), DPI (1 μM), or MitoTEMPO (10 μM) and then incubated with surfactin for 48 h. The cell viability was measured. **(F)** Cells were pretreated with NAC (10 mM) and then incubated with surfactin for 48 h. The caspase activity was analyzed. In addition, SCC4 cells were pretreated with NAC and then incubated with surfactin for 24 or 48 h. The protein expression of cleaved PARP was determined. Data are expressed as mean±S.E.M. of three independent experiments. *P < 0.05; #*P* < 0.01, as compared with control (A, C). *P < 0.05; #*P* < 0.01, as compared with the cells exposed to surfactin alone (B, D, E, F).

Generation of ROS by diverse anti-cancer drugs or phytochemicals has been closely related with the induction of apoptosis in cancers. Moreover, we investigated the effect of surfactin on the production of intracellular ROS in these cells. When ROS production was measured after exposure of SCC4 cells to surfactin at different times (1-8 h) by flow cytometric analysis of CellROX Green Reagent fluorescence intensity (Fig. 2A), it was observed that ROS intracellular levels were increased as early as 2 h after exposure. The sources for ROS include NADPH oxidases, mitochondrial electron transport enzymes, xanthine oxidase, cyclooxygenase, lipoxygenase, and uncoupled nitric oxide synthase 20. Here, we showed that ROS production induced by surfactin was reduced effectively by pretreatment with NAC (a thiol-containing antioxidant), DPI (an NADPH oxidase inhibitor), or MitoTEMPO (a mitochondria-targeted antioxidant) in SCC4 cells (Fig. 2B). In addition, we also found that surfactin enhanced NADPH oxidase activity and mitochondrial superoxide production in a time-dependent manner, which were reduced by pretreatment with DPI or MitoTEMPO (Figs. 2C and D). Next, we investigated whether increased production of ROS was critical for surfactin-induced apoptosis, wherein cells were pretreated with NAC, DPI, or MitoTEMPO, and then incubated with surfactin for 48 h. The results in Fig. 2E showed that apoptosis induced by surfactin was reduced in the presence of NAC, DPI, or MitoTEMPO. Finally, we investigated the roles of ROS in surfactin-indcued caspase activation and PARP cleavage. As shown in Fig. 2F, surfactin-induced caspase-3, -7, -9 activation and PARP cleavage were reduced by pretreatment with NAC in SCC4 cells. Thus, these results indicate that surfactin induces apoptosis by a mechanism requiring the increase of intracellular ROS levels in SCC4 cells.

### Surfactin promotes apoptosis via the mitochondrial pathway

**Figure 3:**
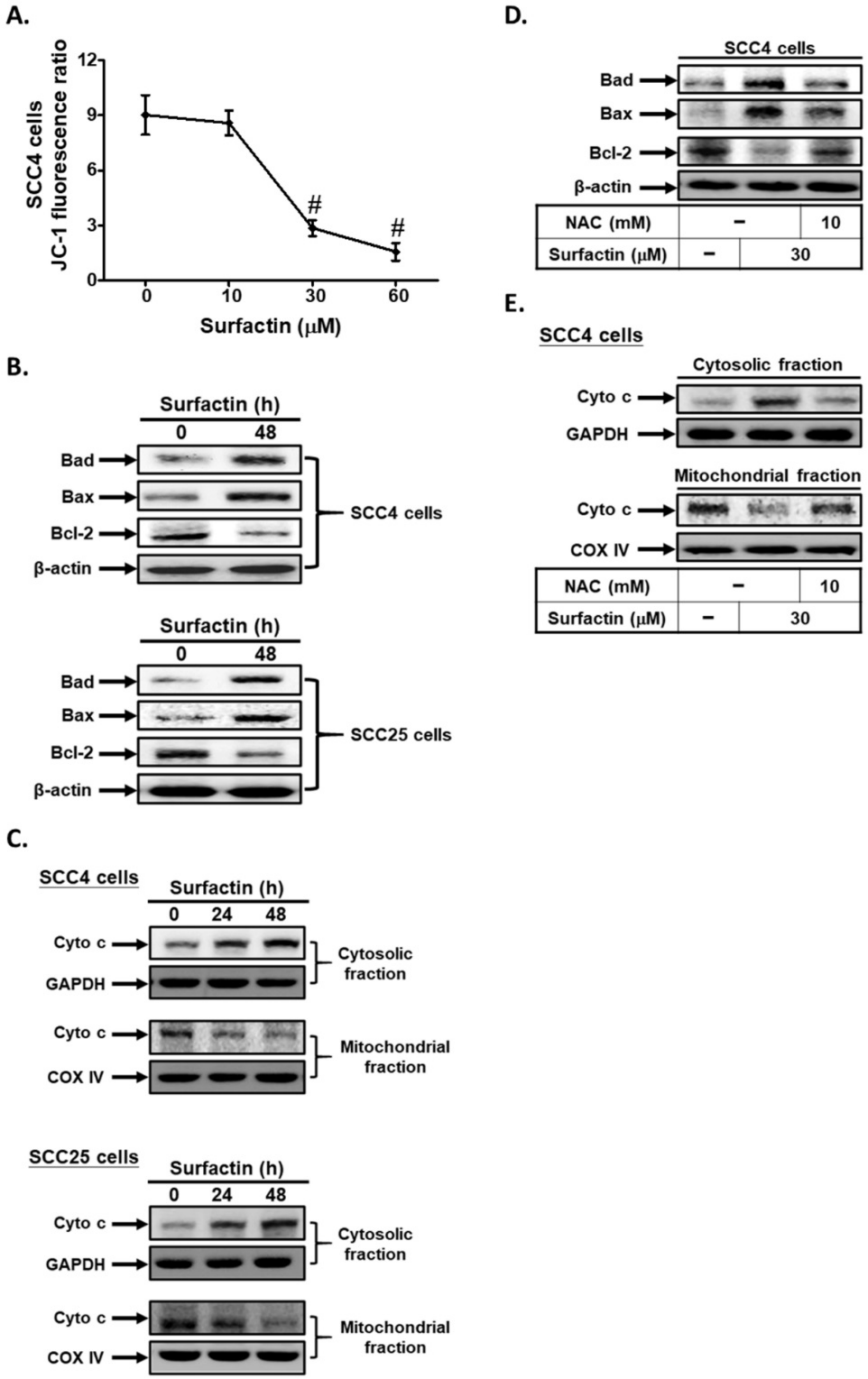
** Surfactin promotes apoptosis through the mitochondrial pathway. (A)** Cells were treated with various concentrations of surfactin for 48 h and then the JC-1 fluorescence ratio was measured. **(B)** Cells were treated with surfactin for 48 h and then the protein expression of Bad, Bax, or Bcl-2 was determined. **(C)** Cells were treated with surfactin for 24 or 48 h and then the cytosolic and mitochondrial fractions were prepared and subjected to Western blot using an anti-cytochrome c antibody. GAPDH was used as a marker protein for cytosolic fractions. COX IV was used as a marker protein for mitochondrial fractions. **(D)** Cells were pretreated with NAC and then treated with surfactin for 48 h. The protein expression of Bad, Bax, or Bcl-2 was determined. **(E)** Cells were pretreated with NAC and then treated with surfactin for 48 h. The cytosolic and mitochondrial fractions were prepared and subjected to Western blot using an anti-cytochrome c antibody. Data are expressed as mean±S.E.M. of three independent experiments. #*P* < 0.01, as compared with control.

Mitochondrial changes, including permeability transition pore opening and the collapse of ΔΨm, result in the release of cytochrome c into the cytosol, which subsequently causes apoptosis by the activation of caspases 21. Here, we used a fluorescent dye JC-1 to detect mitochondrial membrane potential (ΔΨm) in surfactin-treated SCC4 cells. The relative ratio of red and green fluorescence represented the change of mitochondrial membrane potential (ΔΨm). As shown in Fig. 3A, the JC-1 fluorescence ratio significantly reduced at 48 h in a dose-dependent manner. The mitochondrial-mediated pathway of apoptosis is regulated by the Bcl-2 family of antiapoptotic (Bcl-2, Bcl-XL, Bcl-1) and proapoptotic proteins (Bax, Bad, and Bak) 14. Here, we proved that surfactin increased Bad and Bax expression and reduced Bcl-2 expression in SCC4 and SCC25 cells (Fig. 3B). In addition, surfactin was also found to cause a mitochondrial membrane permeability transition, as determined by Western blot of the release of cytochrome c from the mitochondria into the cytosol in cells exposed to the drug for 24 or 48 h (Fig. 3C). Finally, we observed that pretreatment with NAC markedly decreased surfactin-induced Bad and Bax expression and up-regulated surfactin-reduced Bcl-2 expression in SCC4 cells (Fig. 3D). On the other hand, pretreatment with NAC also reduced surfactin-induced the release of cytochrome c from the mitochondria into the cytosol (Fig. 3E). Thus, these data suggest that surfactin induces apoptosis via the mitochondrial pathway in SCC4 and SCC25 cells.

### ROS induces JNK1/2 activation leading to apoptosis

**Figure 4:**
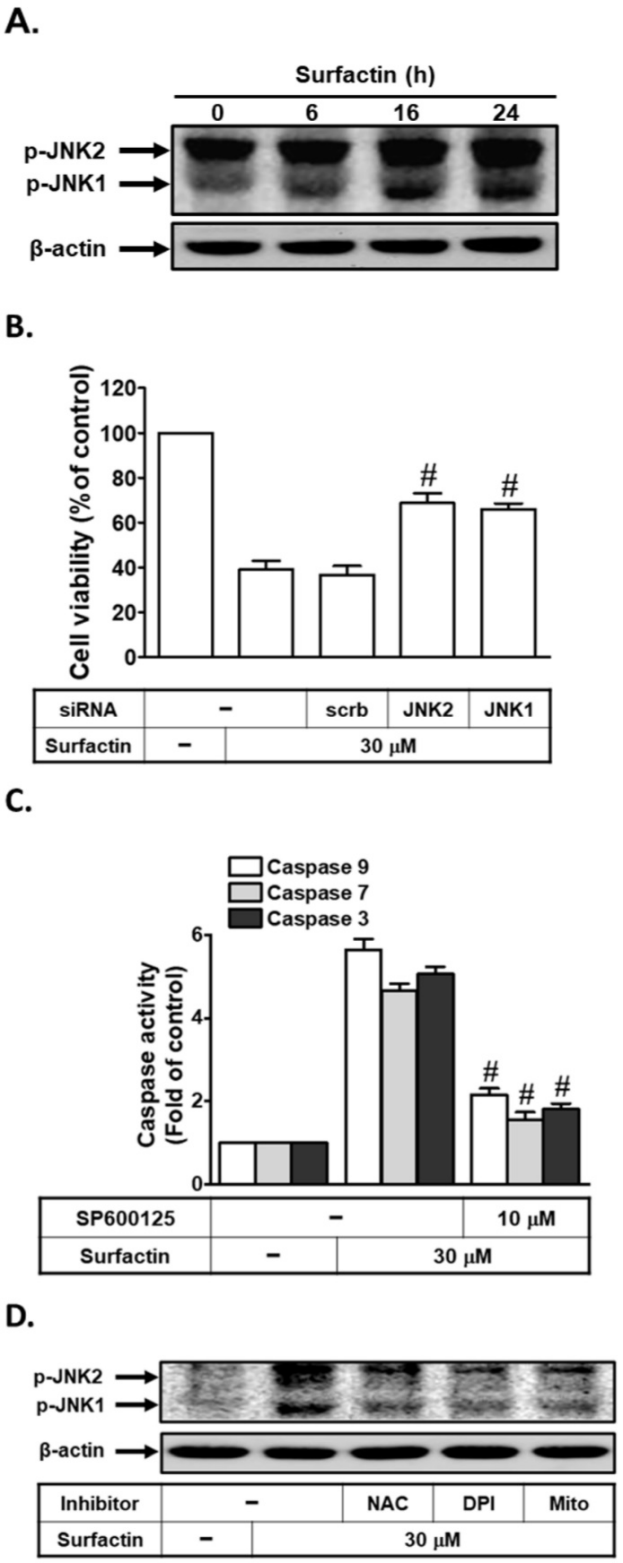
** ROS induces JNK1/2 activation leading to apoptosis. (A)** SCC4 cells were treated with surfactin for the indicated times and then the protein levels of phospho-JNK1/2 were determined. **(B)** SCC4 cells were transfected with siRNA of scrambled, JNK1, or JNK2 and then treated with surfactin for 48 h. The cell viability was assayed. **(C)** SCC4 cells were pretreated with SP600125 for 1 h and then incubated with surfactin for 48 h. The caspase activity was analyzed. **(D)** SCC4 cells were pretreated with NAC, DPI, or MitoTEMPO and then incubated with surfactin for 24 h. The protein levels of phospho-JNK1/2 were determined. Data are expressed as mean±S.E.M. of three independent experiments. #*P* < 0.01, as compared with the cells exposed to surfactin + scrambled siRNA (B) or surfactin alone (C).

Generation of intracellular ROS is known to activate the MAPK pathway with MAPKs being relevant in apoptosis signaling 22,23. Here, phosphorylation of JNK1/2 strongly increased after 16 h and was sustained at 24 h following exposure to surfactin (Fig. 4A). We further investigated whether JNK1/2 was critical for surfactin-induced apoptosis, cells were transfected with JNK2 or JNK1 siRNA and then analyzed for apoptosis after exposure of surfactin for 48 h. The results in Fig. 4B showed that apoptosis induced by surfactin was reduced in the presence of either JNK1 or JNK2 siRNA. In addition, we investigated the role of JNK1/2 in surfactin-induced caspase activation. As shown in Fig. 4C, surfactin-induced caspase-3, -7, and -9 activation were reduced by pretreatment with SP600125 (an anthrapyrazolone inhibitor of JNK1/2) in SCC4 cells. ROS have been shown to regulate JNK1/2 phosphorylation in various cell types 22,23. Here, we found that surfactin-induced JNK1/2 activation was inhibited by preincubation with NAC, DPI, or MitoTEMPO (Fig. 4D). However, SP600125 pretreatment had no effects on surfactin-induced ROS generation and NADPH oxidase activation in SCC4 cells (Supplementary Fig. 2). Thus, these data suggest that surfactin induces apoptosis via the ROS/JNK1/2 signaling pathway in SCC4 cells.

## Discussion

PM exposure increases morbidity and mortality as well as causes chronic lung inflammation and cardiovascular diseases. Recently, Chu et al. proved that Taiwanese men exposed to higher concentrations of PM2.5 have an increased risk of oral cancer 2. The seventh most common cancer in the world is OSCC which has been considered to cause detriment to human health and high mortality 24. Surfactin is a bacterial cyclic lipopeptide generated by *Bacillus subtilis* 15. Recently, surfactin has been shown to suppress cancer progression by cell cycle arrest, apoptosis, growth inhibition, and metastasis arrest 25. The potential mechanisms by which surfactin inhibit human OSCC progression in response to PM still remain unclear. In this study, we found that surfactin decreased the viability of SCC4 and SCC25 cells by induction of apoptosis, which was associated with activation of caspase and PARP cleavage and was regulated by the mitochondrial pathway, as exemplified by mitochondrial depolarization, cytochrome c release, and down-regulation of the antiapoptotic Bcl-2 protein. We also observed that intracellular ROS production appeared essential for the activation of the mitochondrial pathway and the induction of apoptosis after exposure to surfactin. After treatment with surfactin, ROS provided a specific environment that resulted in JNK1/2-induced cell death. These data suggest a potential effect of surfactin in generating ROS and inducing mitochondria-associated apoptosis via prooxidative activity in human OSCC.

Most research in the past had explored the relationship between betel nuts, cigarettes, or alcohol consumption and oral cancer, but few studies have investigated the relationship between air pollution and oral cancer. PM is typically a representative indicator of air pollution 2,4. It does more damage to humans than any other pollutant. The composition of PM is very complicated that includes nitrate, sulfate, ammonia, and so on. Compared to PM10, PM2.5 might cause greater and more severe adverse effects to human health 4. PM2.5 generally penetrates the lung barrier and enters the blood system. Zhang and Li indicated that PM2.5 could induce the cell proliferation, migration, and invasion of human hepatocellular carcinoma (HCC) cell line SMMC-7721 26. *Bacillus subtilis* generates the cyclic lipopeptide surfactin. Its heptapeptide head has two negatively charged amino acid residues, and its tail consists of fatty acid residues 25. Recently, surfactin has been shown to possess some properties including anti-cancer, anti-bacterial, and anti-viral activities 16. Biochemical pathways of apoptosis activation have been extensively studied that can be extra- or intracellular, and caspase-dependent or caspase-independent 19. Caspases are actively involved in inflammatory processes and apoptosis. In this study, we proved that surfactin promoted apoptosis in a caspase-dependent manner in SCC4 and SCC25 cells. Interestingly, we found that surfactin did not affect cell viability of normal human oral keratinocytes and human gingival fibroblasts. Therefore, our results directly or indirectly provide new insight into the mechanisms of surfactin-induced cancer cell death.

Oxygen-free radicals, more generally known as ROS, along with reactive nitrogen species (RNS) are well recognized to play a dual role as both deleterious and beneficial species 27. ROS is characterized to be secondary messengers in intracellular signaling cascades, which induce and maintain the oncogenic phenotype of cancer cells; however, ROS can also induce cellular senescence and apoptosis, in turn, can function as anti-tumorigenic species 28. The sources for ROS include NADPH oxidases, mitochondrial electron transport enzymes, xanthine oxidase, cyclooxygenase, lipoxygenase, and uncoupled nitric oxide synthase 29. Here, we proved that ROS production induced by surfactin was decreased by pretreatment with the NADPH oxidase inhibitor, the mitochondria-targeted antioxidant, or the free radical scavenger in SCC4 cells. On the other hand, surfactin-induced mitochondrial ROS production was attenuated by pretreatment with the NADPH oxidase inhibitor, suggesting that surfactin induced mitochondrial ROS production via the activation of NADPH oxidase in these cells. Finally, we found that surfactin-induced apoptosis, caspase-3, -7, and -9 activation, and PARP cleavage were reduced by preincubation with the free radical scavenger, suggesting that increased generation of ROS was critical for surfactin-promoted apoptosis.

Mitochondria participate in a variety of important physiological and biochemical processes, such as tricarboxylic acid cycle, fatty acid metabolism and oxidative phosphorylation 30,31. Mitochondria are indispensable for energy metabolism, apoptosis regulation and cell signaling 30. Mitochondrial changes, including permeability transition pore opening and the collapse of membrane potential, result in the release of cytochrome c into the cytosol, which subsequently causes apoptosis by the activation of caspases 22. In addition, the mitochondrial-mediated pathway of apoptosis is regulated by the Bcl-2 family of antiapoptotic (Bcl-2, Bcl-Xl, Bcl-1) and proapoptotic proteins (Bax, Bad, and Bak) 19. Here, we found that surfactin induced Bad and Bax expression as well as reduced Bcl-2 expression. In addition, surfactin was also revealed to cause a mitochondrial membrane permeability transition, as determined by Western blot of the release of cytochrome c from the mitochondria into the cytosol in cells exposed to the drug. Moreover, these effects induced by surfactin were decreased by preincubation with the free radical scavenger, suggesting that surfactin induced apoptosis via the ROS-dependent mitochondrial pathway in SCC4 and SCC25 cells.

Generation of intracellular ROS is known to activate the MAPK pathway with MAPKs being relevant in apoptosis signaling 32-34. The generic MAPK signaling pathway is shared by four distinct cascades, including the ERK1/2, JNK1/2, p38 MAPK, and ERK5 23. JNK1/2 pathway is reported to be associated with the cell apoptosis 35. Indeed, we found that surfactin induced JNK1/2 activation in SCC4 cells. In addition, we found that surfactin-induced JNK1/2 activation was inhibited by pretreatment with the NADPH oxidase inhibitor, the mitochondria-targeted antioxidant, or the free radical scavenger, consistent with the results that ROS mediated JNK1/2 phosphorylation in various cell types 36,37. Finally, we demonstrated that surfactin-induced apoptosis and caspase-3, -7, and -9 activation were reduced by JNK1 or JNK2 siRNA and SP600125, suggesting that surfactin induced apoptosis through the ROS/JNK1/2 signaling pathway in SCC4 cells.

**Figure 5:**
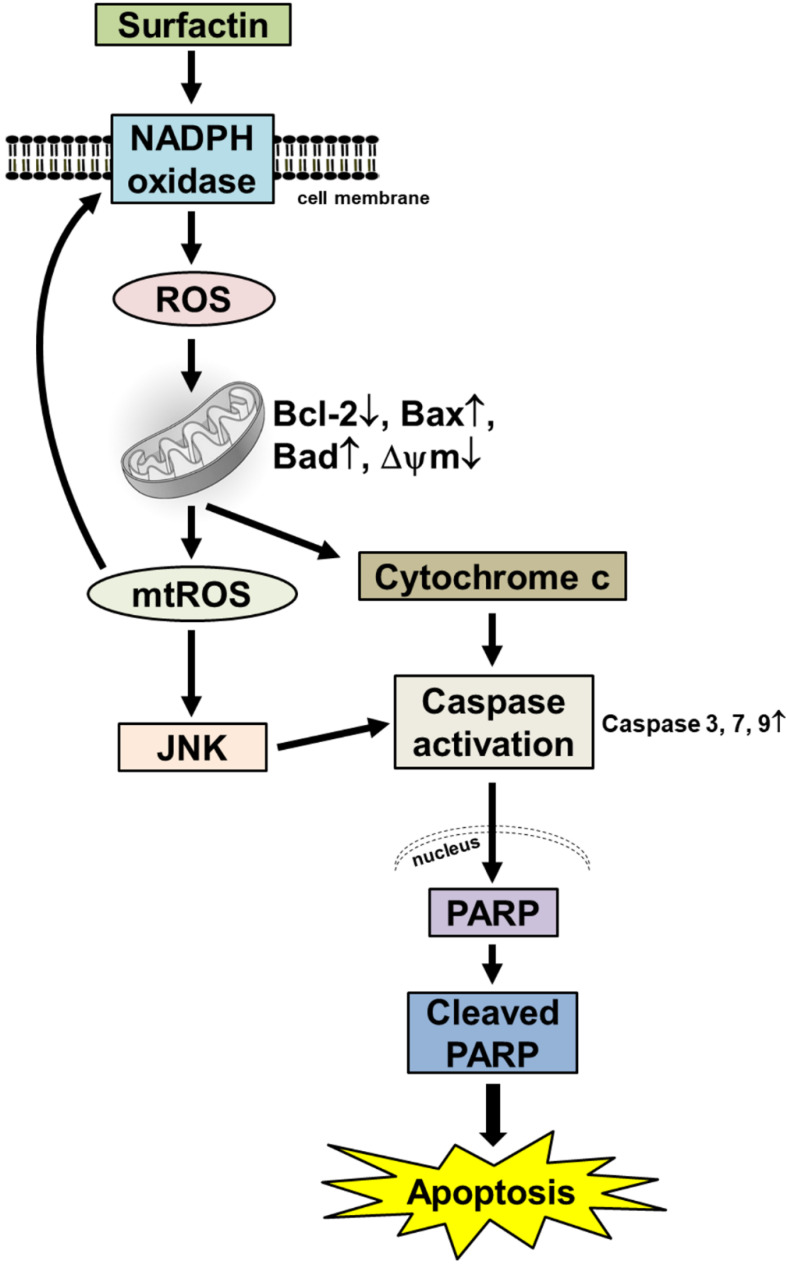
** Proposed model to illustrate the anticancer activity of surfactin in SCC4 and SCC25 cells.** Surfactin reduced the viability of SCC4 and SCC25 cells by induction of apoptosis. Apoptosis promoted by surfactin was associated with activation of caspase and PARP cleavage and was regulated by the mitochondrial pathway, as exemplified by mitochondrial depolarization, cytochrome c release, and down-regulation of the antiapoptotic Bcl-2 protein. Surfactin induced intracellular ROS generation. Intracellular ROS production appeared essential for the activation of the mitochondrial pathway and induction of apoptosis after exposure to surfactin. Oxidative stress due to treatment with surfactin was associated with JNK1/2 activation, as determined by JNK1/2 phosphorylation. After treatment with surfactin, ROS provided a specific environment that resulted in JNK1/2-induced cell death. These findings suggest that surfactin is a potential chemotherapeutic agent for the treatment of oral squamous cell carcinoma.

In summary, as depicted in Fig. 5, our results showed that in SCC4 and SCC25 cells, surfactin-induced apoptosis was linked to the activation of caspase and PARP cleavage mediated by the mitochondrial pathway which demonstrated by mitochondrial depolarization, cytochrome c release, and down-regulation of the antiapoptotic Bcl-2 protein. Intracellular ROS production seems to play a crucial role in the activation of the mitochondrial pathway as well as the induction of apoptosis after exposure to surfactin. Oxidative stress due to treatment with surfactin was associated with JNK1/2 activation. After treatment with surfactin, ROS generated a specific environment that lead to JNK1/2-induced cell death. These findings collectively suggest that surfactin is a potential chemotherapeutic agent for the treatment of human OSCC.

## Supplementary Material

Supplementary figures and tables.
