# Supplementary material for: Surfactin from Bacillus subtilis induces apoptosis in human oral squamous cell carcinoma through ROS-regulated mitochondrial pathway
Source: J Cancer. 2020 Oct 21;11(24):7253–63. doi: 10.7150/jca.50835 (PMC7646178; doi:10.7150/jca.50835)
Supplement: Supplementary file 1 — Supplementary figures and tables. [file jcav11p7253s1.pdf]

Supplementary Fig. 1

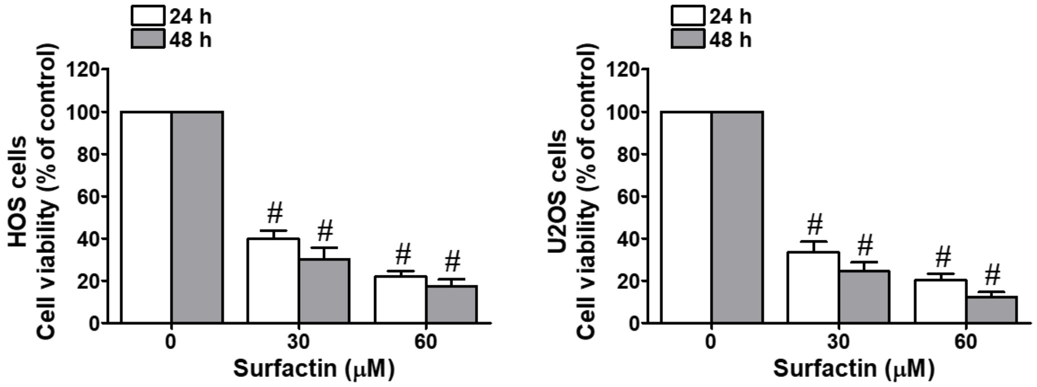

Supplementary Figure 1. Surfactin decreases cell viability in human osteosarcoma cell lines (HOS and U2OS). Cells were treated with various concentrations of surfactin for the indicated times and then the cell viability was measured. Data are expressed as mean±S.E.M. of three independent experiments. #*P* < 0.01, as compared with control.

Supplementary Fig. 2

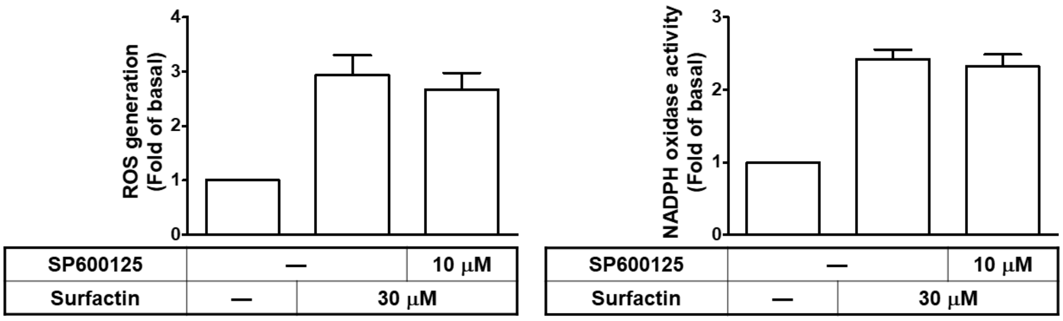

Supplementary Figure 2. SP600125 pretreatment has no effects on surfactin-induced ROS generation and NADPH oxidase activation. SCC4 cells were pretreated with SP600125 (10 μM) for 1 h and then incubated with surfactin for 4 h. The ROS generation and the NADPH oxidase activity were measured.
